# From microscopy to antimicrobial decisions: a clinically grounded roadmap for critical care infectious diseases

**DOI:** 10.3389/frai.2026.1807400

**Published:** 2026-04-13

**Authors:** Hui-Ling Qu, Jia-Nan Li, Yang Gao, Xi-Meng Xu, Xiao-Bin Zhang, Shi-Da Yang

**Affiliations:** 1 Department of Neurology, The General Hospital of Northern Theater Command, Shenyang, China; 2 Department of Neurosurgery, The General Hospital of Northern Theater Command, Shenyang, China; 3 Department of Laboratory Medicine, The People's Hospital of Liaoning Province, Shenyang, China

**Keywords:** antimicrobial stewardship, artificial intelligence, clinical decision support systems, intensive care unit, microbiology diagnostics, sepsis

## Abstract

In the intensive care unit (ICU), antibiotics often begin under extreme uncertainty. Fever, leukocytosis, hypotension, and organ dysfunction may signal bacterial infection, but the same findings are common with aspiration, post-operative inflammation, drug reactions, or sterile systemic inflammation. Cultures take time and their yield falls after antibiotics. Rapid molecular tests and metagenomics can add actionable information, but they also raise the burden of interpreting complex results. Microscopy is one of the few inputs that can shift management within minutes to hours: Gram-stain patterns from positive blood-culture bottles, respiratory specimens, cerebrospinal fluid, and wound material can reshape initial coverage and support early de-escalation when negative. Tissue and cytology help distinguish invasion from key mimics. The gap is consistency-reads vary across observers, workflows differ, and results do not always translate into reliable bedside actions. This review focuses on infectious-disease artificial intelligence (AI) as ICU bedside decision support, rather than as a survey of models. Using ICU sepsis as the primary use case-and neurocritical care as a challenging setting where sedation, brain injury, and noninfectious inflammation often mimic infection-we separate evidence into pathogen signals and host-response signals. We then map both streams to six decisions over the first 72 hours: start now versus pause, choose initial spectrum, reassess and narrow, escalate diagnostics and source control, act on high-risk resistance or invasive pathogens, and stop safely. We summarize where AI is most credible today (Gram-stain assistance, culture-plate triage, urine-culture screening, infection-focused digital pathology, host-response classifiers, and selected metagenomics) and what makes outputs actionable: calibrated probabilities, explicit confidence with safe deferral when uncertain, validation across hospitals and instruments, and endpoints tied to stewardship and safety (time to appropriate therapy, antibiotic days, de-escalation within 72 hours, missed bacteremia). Evidence was updated through February 28, 2026.

## Introduction

Sepsis care starts with a race against time (Singer et al., 2016; Evans et al., 2021; Levy et al., 2017). When shock is likely, delays in effective antibiotics increase harm (Kumar et al., 2006). Clinicians still have to balance this urgency against the real costs of over-treatment: drug toxicity, *Clostridioides difficile*, selection for resistance, and loss of future options. Sepsis remains a leading global cause of death and disability, and antimicrobial resistance increases the stakes for every empiric choice (Tamma et al., 2017; Organization, 2022; Gray et al., 2025).

The core problem is not lack of tests. It is lack of information that can directly guide clinical action in the time window where decisions matter. Blood cultures and standard susceptibility testing are slow. Prior antibiotics lower yield. Many intensive care unit (ICU) patients have more than one process at once, such as aspiration plus atelectasis, or bacterial infection plus sterile inflammation (Evans et al., 2021). In 2024, the World Health Organization published clinical management guidance for sepsis, which reinforces the need for earlier recognition, timely antimicrobials, and safer de-escalation once infection is less likely (Peri et al., 2024; Organization, 2024).

Artificial intelligence can help only when it is anchored to a bedside decision. A probability score that arrives after broad-spectrum therapy has started rarely changes the first choice. It can still matter at the next steps, when teams reassess, narrow, and decide when to stop. In this review, microbiology and host-response findings are treated as time-ordered evidence, not as a single final answer. We focus on six decision points in the first 72 hours: start now versus pause, choose initial spectrum, reassess and narrow, escalate diagnostics and source control, act on high-risk resistance or invasive pathogens, and stop safely.

Beyond prior AI-infection overviews, we emphasize the link from result to action. Consistent with reporting and evaluation guidance for clinical AI, we describe what systems should report so ICU clinicians can judge when to act (Sounderajah et al., 2025; Moons et al., 2025; Collins et al., 2025; Rivera et al., 2020; Vasey et al., 2022; Mongan et al., 2020; Park et al., 2024; Group, 2025). We propose a minimum usable output: a time stamp, specimen quality, calibrated risk for rule-in and rule-out, and an explicit workflow for stewardship review and override.

Review approach and evidence scope. This framework-driven narrative review is organized around ICU bedside decision points during the first 72 hours-whether to start broad therapy, reassess and de-escalate, escalate diagnostics and source control, target high-risk pathogens or resistance, and stop safely. The purpose is to translate diagnostic and AI outputs into clinically actionable stewardship decisions rather than to comprehensively catalog available models. We prioritized high-quality guidelines, multicenter validation studies, and prospective impact evaluations where available; for emerging modalities, we emphasize clinical readiness, safety guardrails, and workflow requirements in addition to reported diagnostic performance. To inform this review, we screened PubMed/MEDLINE and major guideline repositories, prioritizing 2018-2026 publications relevant to ICU sepsis diagnostics, rapid microbiology, host-response testing, metagenomics, digital pathology, and clinical AI reporting standards, using search terms combining ICU/sepsis with microscopy/Gram stain, rapid diagnostics/PCR, metagenomics/mNGS/cfDNA, host response/transcriptomics, digital pathology/foundation models, and stewardship outcomes such as days of therapy, de-escalation, and time to appropriate therapy.

Scope of this review. The primary focus is ICU decision support for suspected bacterial infection or sepsis during the first 72 hours. Host-response tools and metagenomics are discussed as adjuncts to, rather than replacements for, clinical assessment, cultures, imaging, and source control. High-risk contexts (e.g., persistent shock, severe immunosuppression, suspected deep-seated infection, and suspected invasive fungal disease) require conservative thresholds and confirmatory pathways before therapy is changed.

For clarity, we use practical clinical definitions. *Calibration* (probability accuracy) is the agreement between predicted probabilities and observed risk. A *rule-in threshold* is a high-probability cut-off that can justify escalation; a *rule-out threshold* is a low cut-off that can support de-escalation. ICU teams also need a safe 'not sure' state: when inputs are low quality or out of distribution, the system should defer to human review rather than output a confident label in a high-stakes setting (Geifman and El-Yaniv, 2019). Because performance can shift with patient mix, local pathogens, and laboratory methods, monitoring and periodic re-validation are essential (Group, 2025).

For clarity, several terms are used consistently throughout this review. A *72-hour decision point* is the structured reassessment window in which early microbiology and response-to-therapy data can support narrowing, escalation, or safe discontinuation. *De-escalation* refers to narrowing spectrum and/or stopping unnecessary agents while maintaining adequate coverage. A *stop decision* means discontinuing antibiotics when objective safety checks support a non-bacterial diagnosis or clinical resolution. *Escalation* refers to broadening therapy for high-risk resistance or clinical deterioration. *Deferral* is an explicit ‘not sure' state that prompts stewardship review and/or additional testing rather than automatic action.

## A decision-first framework for the first 72 h

In ICU sepsis, the key question is not whether a system is accurate in general (Evans et al., 2021). The question is whether it changes a specific decision at a specific time. The same result can be helpful at hour 2 and irrelevant at hour 26. Time windows should be explicit in every study and in routine-care reports (Rivera et al., 2020; Vasey et al., 2022; Liu et al., 2020).

It helps to frame each system around the ICU decision point it is meant to support:

- Decision Point 1: The first hour: recognize shock, obtain cultures, start broad therapy, and plan source control.
- Decision Points 2 and 3: The next 24–72 hours, when early microbiology arrives, the diagnosis is revisited, and therapy can be narrowed.
- Decision Points 4–6: Escalation for high-risk organisms and resistance, and stopping safely when evidence supports it.

A simple way to keep the review clinically grounded is to pair each decision point with three fields: what data the tool uses, what the result looks like, and what action it supports (Peri et al., 2024). This also makes evaluation clearer. A test that does not change time to appropriate therapy, spectrum, or antibiotic days is unlikely to matter, even with good area under the curve (Timbrook et al., 2017; Vickers and Elkin, 2006). Table 1 maps the six ICU decision points to default actions and safety risks, and Table 2 provides a modality-by-modality view of where each signal fits, what it does well, and where it commonly fails.

**Table 1:** Mapping Evidence to Decisions. Key ICU decision points and how pathogen/host-response evidence support stewardship.

Decision point	Primary evidence available	Model results to report	Stewardship action supported	Key safety risk
1. Start empiric antibiotics (0h)	Vitals, labs, early microscopy, host response	Infection likelihood; Bacterial vs. viral uncertainty	Start or withhold; Choose empiric spectrum	False negative: Delayed therapy leading to mortality in shock.
2. Choose initial spectrum (< 24h)	Syndrome features, imaging, notes, early results	Syndrome probability; Severity risk	Choose regimen; Prioritize monitoring	Overtreatment: Broad therapy driven by low specificity.
3. Reassess & de-escalate (24–72h)	Plate prioritization, prelim ID, clinical trajectory	Rule out significant growth; Probability of response	Narrow or stop within 72h	False reassurance: Automation bias leading to missed infection.
4. Escalate (Shock/Resistance Risk)	Trajectory, repeat microbiology, imaging	Deterioration risk; Missed pathogen signal	Broaden; Add diagnostics; Source control	Alert fatigue: Unnecessary broadening.
5. Target therapy (confirmed)	ID, AST, resistance genes, plate patterns	High-risk pathogen likelihood; Resistance probability	Directed therapy; Infection control steps	Missed rare pathogen or minor resistant subpopulation.
6. Stop therapy safely (>72h)	Trajectory, biomarkers, follow-up cultures	Low relapse risk; Stable/improving signal	Stop antibiotics; Set duration	Premature discontinuation: Leading to relapse or missed infection.

AST, antimicrobial susceptibility testing.

**Table 2:** Modality-by-modality summary: best-fit decision point, strengths, common failure modes, and design requirements.

Modality	Best fit decision point	Strength	Common failure mode	Design requirement
Gram stain microscopy AI	Decision Point 1, 2	Fast, interpretable visual results.	Setting change; Slide quality issues; Confounding.	Quality scoring; safe deferral (“not sure” state); multi-site validation; probability calibration.
Culture plate AI	Decision Point 3, 5	High throughput; Routing negative/mixed plates.	Automation bias; Missed mixed growth.	Audit of auto-verified negatives; Clear escalation policy.
Host response classifiers	Decision Point 1, 3, 6	Early bacterial vs. viral discrimination signal.	Immunosuppression; Co-infection; Timing effects.	Threshold selection; Calibration checks; Subgroup reporting; Net-benefit analysis.
Metagenomic sequencing (mNGS)	Decision Point 5, selected Decision Point 4	Breadth; Detects fastidious organisms.	Contamination; Colonization vs. infection; Over-detection.	Sample-type aware interpretation; Plausibility checks; Adjudicated training sets.
Digital pathology (Large models)	Decision Point 5	Tissue invasion context; Rare organism prioritization.	Artifacts; Bias toward common findings.	Heatmaps; Clinically interpretable evidence; Special stain confirmation; Multi-site validation.
Multimodal fusion	Decision Point 3, 4	Integrates evidence and trajectories.	Confounding; Missing data bias.	Staged, time-aware fusion; Explicit “not sure” flags; Human oversight.

Diagnostic performance must be distinguished from clinical impact. Measures such as AUC, sensitivity/specificity, and calibration are necessary, but for ICU stewardship the key question is whether a result changes management within the relevant time window and improves decision-aligned endpoints—such as time to appropriate therapy, de-escalation within 72 h, days of therapy (DOT), adverse drug events, and recurrence—without increasing missed bacteremia or delays in source control. For a result to be clinically usable, each diagnostic or AI report should specify (i) the clinical question it addresses (rule-in vs rule-out vs triage), (ii) the intended decision point (e.g., start, reassess/de-escalate, escalate, stop), (iii) a calibrated probability or categorical risk band, (iv) an explicit confidence/deferral indicator when uncertainty is substantial, (v) specimen/site context and key limitations (e.g., prior antibiotics, contamination risk), and (vi) a recommended next step or confirmation plan when the output is intermediate or high-stakes.

This is also why authors should report the expected net effect on antibiotic exposure and safety side by side (Vickers and Elkin, 2006). Reports focusing only on discrimination are insufficient. Probability accuracy, decision thresholds, and the clinical trade-off should be presented together (ideally on one page) (Sounderajah et al., 2025; Moons et al., 2025; Vickers and Elkin, 2006).

**Figure 1:**
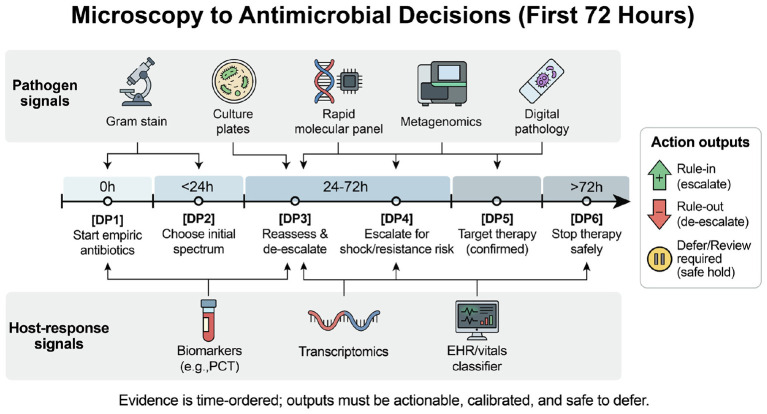
Overview of common pathogen evidence and host-response signals over the first 72 h and beyond (> 72 *h*), highlighting typical windows of availability. Right-side icons indicate stewardship actions enabled by new evidence (escalate/expand coverage, de-escalate/narrow coverage, or hold for review when uncertainty is high).

In day-to-day ICU care, two checks help clinicians decide whether an AI result is worth acting on. First, does the result arrive early enough to change the decision it claims to support? Second, does acting on it have an obvious downside? If the downside is large, such as missing candidemia or delaying source control, then the threshold for safely excluding infection needs to be very conservative and backed by prospective safety data. Figure 1 situates common pathogen-evidence and host-response signals along the first 72 hours, emphasizing when they can realistically influence bedside decisions.

A practical guardrail is a three-zone output: (i) high-confidence rule-in results that justify immediate action (e.g., escalation or early targeted therapy), (ii) high-confidence rule-out results that support narrowing/withholding in low-risk settings, and (iii) an intermediate “deferral” zone that triggers stewardship review, additional sampling, or repeat testing. Thresholds should be chosen to match clinical harm asymmetry and then audited for misses and unintended consequences.

Implementation requires anchoring every system claim to a decision actually made in the first 72 hours (start broad, narrow, stop, or escalate). If a signal cannot arrive in time, it acts as a reassessment system, not an early-decision system. Furthermore, rule-in and rule-out thresholds need to be chosen in advance, and audits should focus on what happens to patients near these thresholds.

## Rapid specimen signals that can change early coverage

### Gram stain: still valuable, still variable

Within minutes, a good Gram stain can answer two bedside questions: is there likely a bacterial burden, and what broad class is present. In septic shock this can justify early coverage for Gram-negative rods, anaerobes, or yeast, while cultures and susceptibilities are pending (Evans et al., 2021; Kumar et al., 2006). The limitation is not biology. It is variation. Slides vary by stain quality, specimen quality, and reader experience.

AI may reduce inter-reader variation when it is designed as a second reader, not a replacement. The strongest evidence is for automated interpretation of Gram stains from positive blood cultures and direct smears, where the field is relatively standardized (Smith et al., 2018; Kim et al., 2022; Hardy et al., 2020). For ICU use, we suggest a three-part output: (i) a morphology class with a calibrated probability, (ii) a specimen-quality score, and (iii) a safe-deferral flag when the slide is too poor or too mixed to trust. This matters because mixed growth and contaminant patterns are common in the ICU. False certainty can drive unnecessary escalation.

To be clinically usable, Gram-stain tools must work across hospitals, staining protocols, microscopes, and routine laboratory workflows (Smith et al., 2018; Kim et al., 2022; Hardy et al., 2020; Shwetha et al., 2025). A system that depends on a single stain or camera setup is unlikely to generalize. Reports should include the real range of slide quality and mixed morphologies-not only clean teaching examples.

Workflow details matter as much as model metrics: which fields are rechecked, time from slide read to result release, escalation pathways, and how discordant reads are handled, because downstream impact depends on workflow as well as accuracy (Peri et al., 2024; Timbrook et al., 2017; Hardy et al., 2020). Whole-slide approaches may improve scalability by reducing the need for patch-level manual annotation (McMahon et al., 2025).

Clinically, Gram-stain assistance systems are among the most deployment-ready infection-AI applications when they provide specimen-quality scoring, calibrated probabilities, and an explicit deferral state, and when implemented as a second reader within laboratory QA workflows.

### Urine and respiratory specimens: prioritization, not magic

Many ICU cultures are ordered to rule out infection rather than to confirm it. Urine cultures in catheterized patients and respiratory cultures in ventilated patients often reflect colonization. AI can help most when it reduces low-value work: screening out low-quality samples, flagging likely contamination, and prioritizing samples with high bacterial burden for rapid review.

In clinical practice, microscopy AI functions best as a prioritization tool: it is most useful when it helps choose a safer initial spectrum or prompts a rapid re-sample. Clinicians should request the image, not only the label. Mixed morphologies and poor-quality fields are where mistakes start. Furthermore, a ‘review required' signal is a feature, not a failure; a safe hold is safer than false certainty.

The clinical test is simple: Does the tool reduce unnecessary antibiotics or unnecessary cultures without increasing missed bacteremia or delayed appropriate therapy (Peri et al., 2024)? Studies should predefine these safety endpoints and audit the high-risk misses, not only report sensitivity (Tamma et al., 2017; Vickers and Elkin, 2006).

### A practical reporting template for early microbiology

Early microbiology reports should read like an ICU note rather than a laboratory printout. They should state the specimen collection time, the time the slide was read, and which decision point the result can still influence. For any score, reports should include practical rule-in and rule-out cut-offs and indicate whether predicted probabilities are calibrated (for example, with a simple reliability plot) (Sounderajah et al., 2025; Moons et al., 2025). Reports should also communicate confidence in plain language and name the stewardship and laboratory-review clinical workflow. A clinician-facing report template can make uncertainty and follow-up actions explicit while preserving traceability for later audit (Sounderajah et al., 2025; Moons et al., 2025; Mongan et al., 2020; Park et al., 2024).

## Culture plates and laboratory workflow: where speed really comes from

Turnaround time in microbiology is often limited by care workflow more than by AI tools. A system can only help the bedside if the lab process can deliver an actionable result early in the clinical course. That means fast specimen transport, clear prioritization rules, and reliable communication back to the ICU.

### Pre-analytics: specimen quality is the first model

In ICU practice, the earliest failure often happens before the AI tool runs. Poor sampling, delays to the lab, and mislabeled specimens create noise that no system can repair. For microscopy and molecular assays, studies should report how specimens were collected, how quickly they were processed, and how often samples were rejected for quality.

Approaches that can score sample quality and trigger a re-sample request can deliver more value than marginal gains in accuracy. For respiratory samples, mixed flora and low-quality sputum are common. For line-associated infections, contamination is common. A bedside-ready system should expose these risks explicitly, not hide them behind a single label. This is a clinical safety requirement, not a technical detail.

### Communication: the result has to land in the right place

Even a fast lab result can be wasted if it is not delivered to the decision maker. ICU clinical workflows differ (Peri et al., 2024; Timbrook et al., 2017). Some units rely on stewardship pharmacists. Others rely on intensivists. Many rely on a mix. Papers should state who received the alert, how quickly they responded, and whether the recommendation was accepted. This is part of the intervention and it should be stated. Digital microbiology makes culture work more like a continuously updated process (Signoroni et al., 2023; Bard et al., 2024; Faron et al., 2020; Bailey et al., 2019). Plate images can be captured at defined time points. Plate-image datasets can also be expanded with synthetic generation and style-transfer augmentation to support model development (Pawłowski et al., 2022). AI can prioritize plates that are negative, mixed, or need early colony selection. This can free technologist time for the plates that matter and can shorten time to identification and susceptibility in practice, even if the tool is not perfect.

Clinically, culture-plate imaging and triage are plausibly high-impact for turnaround time, but bedside benefit depends on laboratory process redesign and audited auto-verification policies for negative plates and mixed growth.

## Host response models: useful if they answer a bedside question

### What host-response tools can and cannot claim

Host response approaches try to answer a different question than microbiology (Shean and Greninger, 2019). They ask whether the physiology and immune pattern looks more like bacterial infection, viral infection, or sterile inflammation. In the ICU this is attractive because cultures are often negative and antibiotics are often started before samples are taken.

Two families matter to clinicians and should be described separately. Transcriptomic signatures measure host ribonucleic acid (RNA) expression and can help separate bacterial from viral patterns, but they add laboratory cost and may not return within the first decision window (Shean and Greninger, 2019; Scicluna et al., 2025; Stevens and Harris, 2025). Phenotype tools built from routine electronic health record (EHR) data and vital signs can run continuously and cheaply, but they are more vulnerable to confounding from sedation, surgery, immunosuppression, and local care patterns. This limitation is not theoretical: external validation of a widely implemented proprietary EHR sepsis prediction model found poor discrimination and calibration, which shows why EHR-only tools need independent validation and careful claims (Wong et al., 2021).

The risk is over-claim (Moons et al., 2025). A host response signal can be strongly associated with infection yet still be unsafe for stopping therapy in shock, neutropenia, or deep-seated infection (Scicluna et al., 2025; Stevens and Harris, 2025). ICU papers should state where the tool is intended to be used and where it needs to defer. They should also show performance in hard subgroups such as prior antibiotics, renal failure, steroid exposure, and immunosuppression.

Clinically useful results are simple. Provide a calibrated probability of bacterial infection. Provide an explicit confidence flag. Provide a recommended action only when the risk crosses a pre-defined threshold and the patient meets safety criteria for that action. Otherwise, the result should be framed as one piece of evidence for reassessment, not as an order. A recent systematic review and meta-analysis summarizes diagnostic accuracy across commercial platforms and highlights where resistance-gene calls are reliable and where confirmatory work is still needed (Wang et al., 2025).

**Figure 2:**
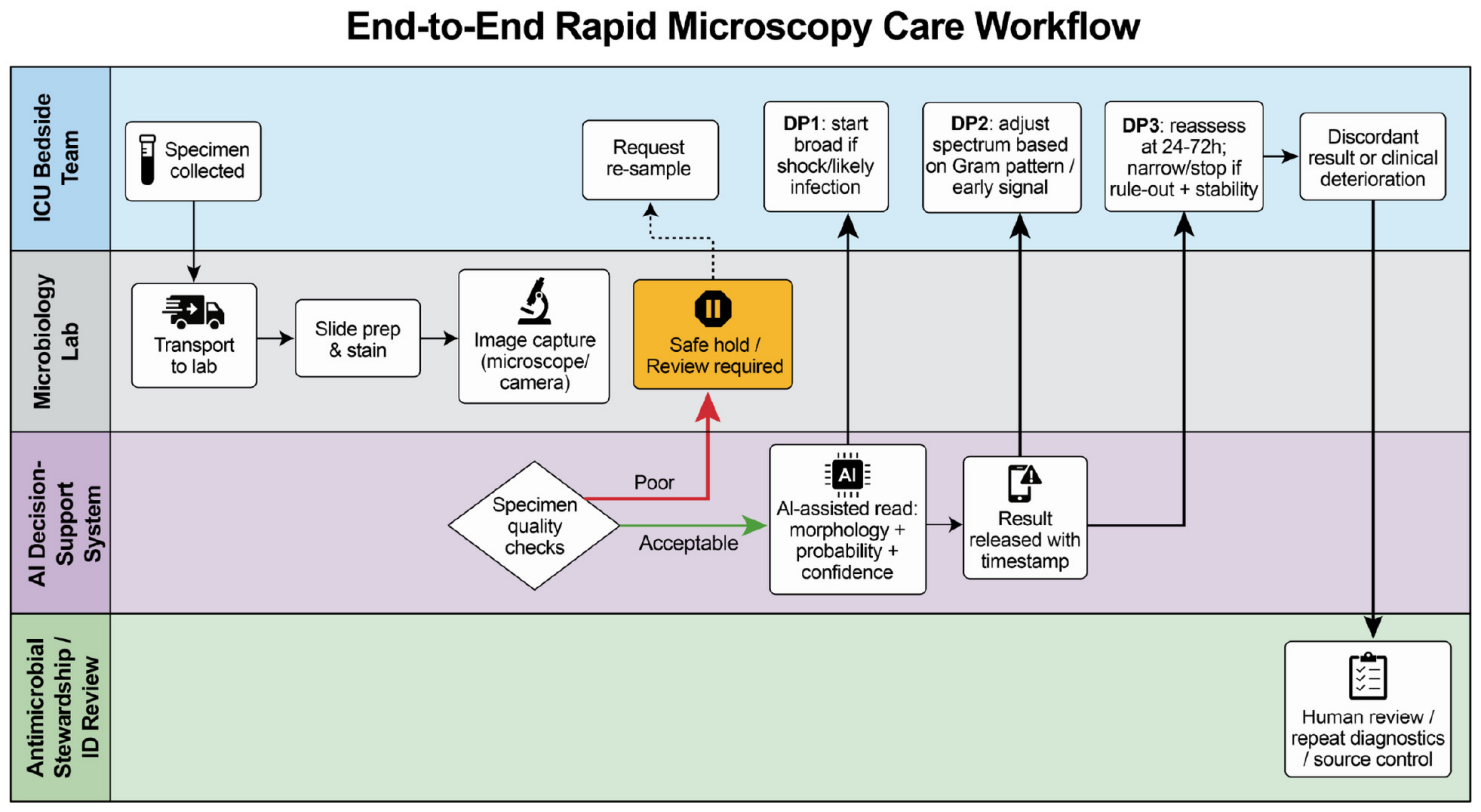
End-to-end rapid microscopy workflow for ICU decision support, linking specimen collection, slide preparation and image capture, specimen-quality checks, results with a safe-hold (review-required) state, and stewardship actions across decision points 1–3.

Bacterial-versus-non-bacterial guidance should be separated from severity prediction. They answer different clinical questions. Host-response signals are most useful for supporting de-escalation when the clinical trajectory and microbiology are concordant. Before adopting a single threshold, studies should report performance in key subgroups (e.g., immunosuppression, renal failure, and diverse populations). In ICU sepsis, the goal is not to automate decisions. The goal is to surface the first reliable signal and update it as new evidence arrives-mirroring how bedside hemodynamics are reassessed. Figure 2 summarizes an end-to-end care workflow, from specimen collection to a stewardship action tag.

In practice, host-response tools are best used as adjuncts in clinically stable patients to support reassessment and potential de-escalation when combined with objective safety checks and follow-up plans. They should not be used alone to stop therapy in persistent shock, severe immunosuppression, suspected deep-seated infection, or suspected invasive fungal disease; in these contexts, confirmatory evidence and a predefined rescue pathway are required.

At present, host-response tools are best positioned to support reassessment and de-escalation in stable patients; broad claims for stop decisions in high-risk physiology should be reserved for settings with prospective safety evaluation and explicit rescue pathways.

### Biomarkers and stewardship: the procalcitonin lesson

Procalcitonin (PCT) is a useful reminder that biomarkers only help when they are tied to a clear bedside plan. In trials that used explicit stopping rules and scheduled reassessment, procalcitonin-guided strategies reduced antibiotic exposure without obvious harm in selected ICU populations (de Jong et al., 2016). The same principle applies to AI: the tool alone does not change care. What changes care is the workflow around it-who receives the result, what decision it is meant to support, what requires confirmation, and how safety is audited (e.g., missed bacteremia, relapse, or inappropriate de-escalation).

## Metagenomics and rapid molecular tests: signal with new burden

### Where rapid molecular tests help today

Rapid molecular panels from positive blood cultures have a clear clinical niche (Timbrook et al., 2017; Wang et al., 2025). They can identify common pathogens and key resistance genes hours to a day earlier than standard methods. When paired with a rapid stewardship response, these tests shorten time to effective therapy and can reduce broad-spectrum exposure (Peri et al., 2024).

The key operational point is pairing. A panel alone does not change care. A panel plus a call to the ICU, plus a clear recommendation, often does. Studies should report the stewardship bundle and the response time, not only the assay measures (Peri et al., 2024). Stewardship frameworks such as the Infectious Diseases Society of America (IDSA) guidance help define who acts on results, how recommendations are communicated, and how harms are tracked (Curren et al., 2022; Barlam et al., 2016). For suspected hospital-acquired or ventilator-associated pneumonia, rapid syndromic polymerase chain reaction (PCR) testing at the bedside has now been evaluated in a pragmatic multicenter randomized trial (INHALE WP3) (Enne et al., 2025). In-ICU PCR improved antibiotic stewardship at 24 hours, but did not demonstrate non-inferiority for clinical cure at 14 days. Speed helps only when it is paired with clear pathways, explicit safety checks, and auditing for unintended harm.

Rapid syndromic PCR and blood-culture molecular panels are clinically ready when they are paired with a time-bounded stewardship response; the diagnostic itself should be treated as incomplete without the response workflow.

In routine practice, data should not be pooled across studies with different time windows. Preferred outcomes reflect action at the bedside: time to appropriate therapy, de-escalation, antibiotic days, and safety. The care workflow is part of the intervention; without the stewardship response, the ‘test' is incomplete.

### Clinical metagenomics: where it helps and where it misleads

Metagenomics can detect organisms that cultures miss. It can also detect deoxyribonucleic acid (DNA) that does not represent invasive infection. In the ICU, both are common. The practical question is whether metagenomics changes treatment in a way that improves outcomes without increasing harm. This is especially relevant for hard-to-diagnose syndromes such as meningoencephalitis, where unbiased sequencing has changed diagnostic yield in practice (Wilson et al., 2019).

Recent multicenter studies report high detection rates for circulating microbial cell-free DNA sequencing in sepsis and suggest that results could change therapy in a meaningful fraction of cases. However, most evidence remains observational, so these studies indicate where testing may matter rather than proving patient benefit. For example, the multicenter NextGeneSiS study reported higher pathogen detection than blood culture in sepsis and identified cases where an expert panel would have adapted therapy. Another large multicenter sepsis cohort linked metagenomic next-generation sequencing (mNGS) to antibiotic-management changes and reported signals relevant to real-world adoption (Brenner et al., 2025; Xu et al., 2025).

To make metagenomics clinically usable, reports should separate three concepts: plausibility (does the organism fit the syndrome?), actionability (is there a treatment change?), and urgency (does the change need to happen now?). A system that does not state these clearly will push unnecessary escalation.

Interpretation checklist (metagenomics/cfDNA): in practice, six questions should be addressed before acting. Is the organism clinically plausible for the syndrome and site? Is it concordant with imaging, host factors, and exposure history? Is it actionable now, or does it mainly generate hypotheses? How likely are contamination or colonization, especially with reagent or environmental signals and low-level translocation? What confirmatory test will be paired with any high-stakes action? What clinical deterioration or discordant data would trigger escalation despite a negative or low-confidence result?

A useful safety rule is to pair every high-stakes action with a confirm step. If the tool suggests a rare invasive fungus, plan early antifungal therapy plus a path to confirmation, such as bronchoscopy, targeted polymerase chain reaction (PCR), antigen testing, or biopsy (Brenner et al., 2025; Xu et al., 2025). If the tool suggests a low likelihood of bacteria, pair de-escalation with close reassessment and a documented rescue plan. This keeps the clinical workflow transparent and reviewable (Vasey et al., 2022; Group, 2025).

Metagenomics also requires a disciplined approach to false positives. Background environmental DNA, low-level translocation, and reagent contamination can all appear as detections. In ICU practice, low-abundance results are best treated as hypotheses until they fit the syndrome, imaging, and repeat testing. A useful report presents organism abundance, common assay contaminants, and whether the signal appears in negative controls. If those details are missing, stewardship should slow down rather than speed up.

Metagenomics is most useful as a targeted, second-line test in high-risk, culture-negative syndromes where decisions are otherwise blind. Reports should prioritize plausibility, actionability, and urgency-an unfiltered organism list is not a clinical report. Any high-stakes action triggered by sequencing should have a confirm-and-rescue clinical workflow. Ownership needs to be explicit: who receives the result, who responds, and how disagreements are documented. Alerts should be proportionate to urgency-interruptive only when delay changes outcome; otherwise, route to a review queue. Finally, post-deployment monitoring for drift and unintended harm is as important as tool accuracy (Group, 2025).

At present, mNGS/cfDNA is best used selectively in high-risk, culture-negative, high-stakes syndromes with confirmation plans; broad first-line use remains premature in many ICUs because of uncertainty about actionability, contamination risk, and limited prospective impact evidence.

Time still matters more than sensitivity. A perfect result at day 5 is too late for the first decisions. It can still matter for narrowing, stopping, and source control. This is why the intended decision point and the turnaround time need to be reported together in every metagenomics study. A brief privacy and oversight statement is worth adding. Many metagenomic clinical workflows include host DNA depletion or computational host-read subtraction. Human reads are hard to eliminate completely. Programs should state what human sequence data are retained, what are discarded, and how access is controlled (Vasey et al., 2022; Group, 2025).

## Infection focused digital pathology and large pre-trained models

### What large pre-trained models change for infection pathology

Digital pathology matters for infection because tissue is often the definitive diagnostic arbiter when blood cultures and imaging are equivocal. In the ICU, a lung biopsy can separate invasion from colonization. A bone biopsy can confirm osteomyelitis. A brain biopsy can rule out mimics such as tumor or vasculitis. The bottleneck is specialist time and the delay between sampling and a confident read.

General-purpose foundation models are trained on very large collections of whole-slide images (Huang et al., 2024; Chen et al., 2024b; Alfasly et al., 2025; Neidlinger et al., 2025). They learn reusable, general histology features. That is especially useful in infection, where the problem has a large number of rare but clinically important pathogens. Rare organisms and unusual host patterns are exactly where labels are scarce. In principle, a foundation model can adapt with few examples, making rare-pathogen detection and prioritization more realistic than building a bespoke system for every organism.

Clinically, the first goal is not to replace the pathologist. The goal is to speed up and standardize review: highlight suspicious fields, prompt appropriate special stains, and reduce misses on low-prevalence findings. Early work in narrow targets, such as *H. pylori*, illustrates a safer pattern: explicit reference standards, human confirmation, and transparent confidence around borderline cases (Zhou et al., 2020).

Reports should be specific about failure modes. Infection slides are messy. Necrosis, cautery, crush artifact, and treatment effect can dominate the image. Report safe hold rates, what triggers a ‘review required' flag, and what the tool does when the slide quality is poor or the tissue does not contain the target compartment.

### Multimodal pathology: linking slide, report, and microbiology

Multimodal pathology links slide images with text reports, microbiology, and clinical phenotypes (Chen et al., 2024b; Lu et al., 2025). This matters in infection because morphology alone is often non-specific. A neutrophilic abscess can reflect bacteria, fungi, sterile injury, or sampling error. Context reduces false certainty while keeping the evidence traceable.

For ICU trust, results should read like a clinical report. Show where the evidence is on the slide. Summarize the key pattern in one sentence. List the leading competing explanations and the next test that would reduce its confidence. This ‘what we saw, what it could mean, what to do next' format is easier to act on than a probability number alone.

At the bedside, infection pathology benefits first from speed and consistency: prioritization, field-of-view highlighting, and stain recommendations. Few-shot adaptation matters because infection is a long-tail problem. Rare pathogens will never have large labeled datasets. Multimodal results should read like a consult note: what is seen, what it could mean, and what additional tests would reduce uncertainty.

At present, digital pathology AI is most immediately useful for triage, region-of-interest highlighting, and stain recommendation support; autonomous infection diagnosis should be avoided outside carefully validated, pathologist-in-the-loop workflows.

## Evidence synthesis that respects time

Most AI reviews pool studies as if they were static diagnostic tests. ICU infection approaches are not static. They run inside clinical workflows. They change with staffing, instruments, pathogens, and prescribing. Evidence synthesis therefore has to respect time and setting. Heterogeneity is expected in ICU infection studies. Different patient-mixes, different contamination rates, and different lab platforms change both prevalence and error profiles. Meta-analyses should therefore report context variables, not only summary sensitivity and specificity. Where pooling is unsafe, narrative synthesis with explicit decision-point framing is more honest and more useful for clinicians.

Time-aware evidence synthesis starts by grouping approaches by when they can realistically report. Bedside and early laboratory signals belong in the first hour. Rapid panels from positive cultures often land in the 12-24 hour window. Metagenomics and pathology frequently belong to 24-72 hours. Pooling across these windows hides the key clinical question: does the result arrive before the decision it aims to change? Future systematic reviews should stratify by time-to-action and by antibiotic stewardship clinical workflow, not only by technical approach.

Evaluation requires three steps:

Match evaluation to the decision point, and pre-specify outcomes that reflect the claimed clinical use case (Vasey et al., 2022; Mongan et al., 2020; Park et al., 2024). If a system claims it can support early narrowing, the outcome should be de-escalation within 48 to 72 hours, not overall hospital mortality. If it claims to improve initial coverage, the outcome should be time to appropriate therapy and missed resistant infection (Levy et al., 2017; Kumar et al., 2006), not only area under the curve; decision-curve or net-benefit analyses can make these trade-offs explicit (Vickers and Elkin, 2006). Report net benefit, not only accuracy. A small change in false negatives can be catastrophic in septic shock, while a small change in false positives can drive unnecessary escalation. Net-benefit curve analysis and pre-defined clinical thresholds help readers judge whether benefits outweigh harms in a given setting (Vickers and Elkin, 2006). Prefer designs that survive real world shifts. Retrospective validation is a start. Prospective shadow evaluations, where results are captured but not shown to clinicians, are stronger. Impact studies with stewardship pathways are stronger still. Post-use monitoring in routine care should be routine, because performance changes over time are expected, not rare (Vasey et al., 2022; Mongan et al., 2020; Park et al., 2024; Group, 2025).

**Figure 3:**
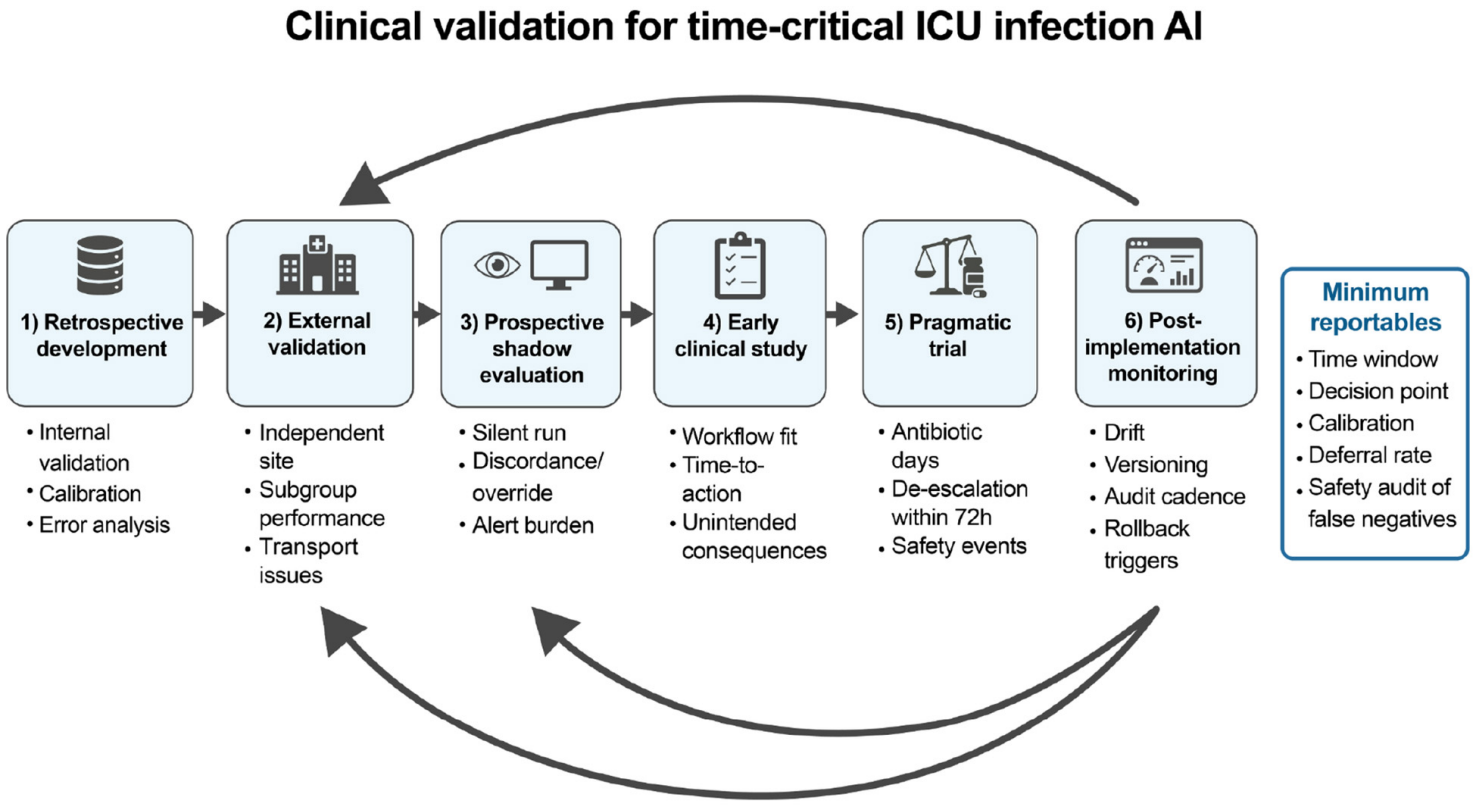
Clinical validation steps for time-critical ICU infection approaches, from retrospective development to external validation, prospective shadow evaluation, early clinical study, pragmatic trial, and post-implementation monitoring. Minimum reportables for each stage are summarized in Table 3.

Figure 3 summarizes this clinical validation approach. Valid clinical interpretation requires reporting the time window, probability accuracy, failure modes, and deferral rates. If these are not reported, clinical interpretation is limited (Sounderajah et al., 2025; Moons et al., 2025; Collins et al., 2025; Rivera et al., 2020; Vasey et al., 2022; Liu et al., 2020). Table 3 translates this into a practical set of clinical validation steps that readers can apply across modalities.

**Table 3:** Evaluation and clinical validation steps: minimum evidence to report at each stage and the most relevant standards.

Stage	Goal	Minimum evidence to report	Helpful Standard
Retrospective development	Feasibility and internal validation	Describe the cohort, inclusion criteria, and reference standard; report discrimination and probability calibration; review common errors with clinical adjudication.	PROBAST+AI; TRIPOD-LLM
External validation	Generalization across sites	Test on at least one independent site; report performance and probability calibration; describe transport issues and missing data; report key subgroups (e.g., immunosuppression).	STARD-AI; TRIPOD-LLM
Prospective shadow evaluation	Safety and drift before changing care	Report discordance and override rates; track change over time; pre-specify review of false negatives; quantify alert burden (alerts per 100 patient-days) and alerts per meaningful action (NNA).	DECIDE-AI
Early clinical study	Workflow fit and safety	Usability and workload; time to action; adherence to the stewardship clinical workflow; early safety signals and unintended consequences.	DECIDE-AI
Pragmatic trial	Effect on antibiotic exposure and safety	Days of therapy and spectrum score; time to appropriate therapy; patient-centered safety outcomes (missed bacteremia, relapse); equity analyses where feasible.	CONSORT-AI; SPIRIT-AI
Post-implementation monitoring	Sustained performance in routine care	Version control; ongoing probability calibration checks; drift monitoring; audit cadence; clear criteria to pause, retrain, or roll back when performance degrades.	Local oversight

NNA, number needed to alert (alerts per meaningful action).

## ICU implementation: what changes at the bedside

### Who does what: workflow and governance

Successful implementation requires clear roles before software goes live. Each ICU deployment should have a named owner and a backup, typically a critical care lead paired with microbiology or stewardship. Teams should define who receives results, who can override them, and how disagreements are documented (Vasey et al., 2022; Group, 2025).

Notification should match urgency without creating noise. A practical approach is tiered routing: high-confidence, high-risk findings trigger immediate alerts; lower-confidence results route to stewardship or laboratory review; and uncertain cases trigger a clear “review now” process (Canovas-Segura et al., 2023). In ICU sepsis, alarm fatigue is real, so alerts should be timed and framed around the moment they can change care (Goldart et al., 2024).

### Managing alerts: timing and trust

The notification strategy is as important as the AI tool. A tiered alert system helps prevent alarm fatigue. High-confidence findings involving high-risk pathogens should trigger immediate, interruptive alerts to the bedside team. In contrast, lower-confidence or ambiguous results should be routed to a stewardship or laboratory review queue for expert verification. This approach ensures that bedside clinicians are interrupted only when immediate action is required. Human factors play a significant role. Over-trust and avoidance often coexist in the same clinician. Transparent reports that show the evidence driving the call help reduce over-reliance. An important safety rule applies: if the AI suggests a low likelihood of infection but the patient remains in shock, therapy should not be withheld. Because over-trust can lead to unsafe de-escalation, a low-risk output should trigger structured reassessment and require alignment with clinical stability and negative microbiology before antibiotics are narrowed or stopped (Abdelwanis et al., 2024).

### Making the business case: economics

Economic viability determines whether a system reaches the bedside. A reliable business case separates one-time infrastructure costs (EHR integration, staff training) from recurring per-test costs (reagents, compute). The value proposition should be tied to measurable offsets in resource use. Relevant endpoints include antibiotic days, spectrum scores, time to appropriate therapy, ICU length of stay, and downstream utilization (Peri et al., 2024; Timbrook et al., 2017; Wang et al., 2025; Brenner et al., 2025; Xu et al., 2025). Avoiding adverse antibiotic events-including *C. difficile* infection and drug toxicity-can also contribute. Cost-effectiveness analyses are emerging for targeted applications, such as metagenomic testing in postoperative central nervous system infection (Tian et al., 2025). Where evidence is mixed, we use conditional language (may/can) and emphasize workflow dependence.

Evidence suggests that rapid diagnostic tests do not create economic value in isolation. Value is generated only when results are paired with a stewardship intervention that accelerates clinical action. For expensive modalities like metagenomics (mNGS), the most favorable economic profile is found in selective use for high-risk, culture-negative patients, where the test can replace days of ineffective broad-spectrum therapy and repeated non-diagnostic testing. Recent data from pilot studies support the cost-effectiveness of this targeted strategy.

### Monitoring safety and equity

**Figure 4:**
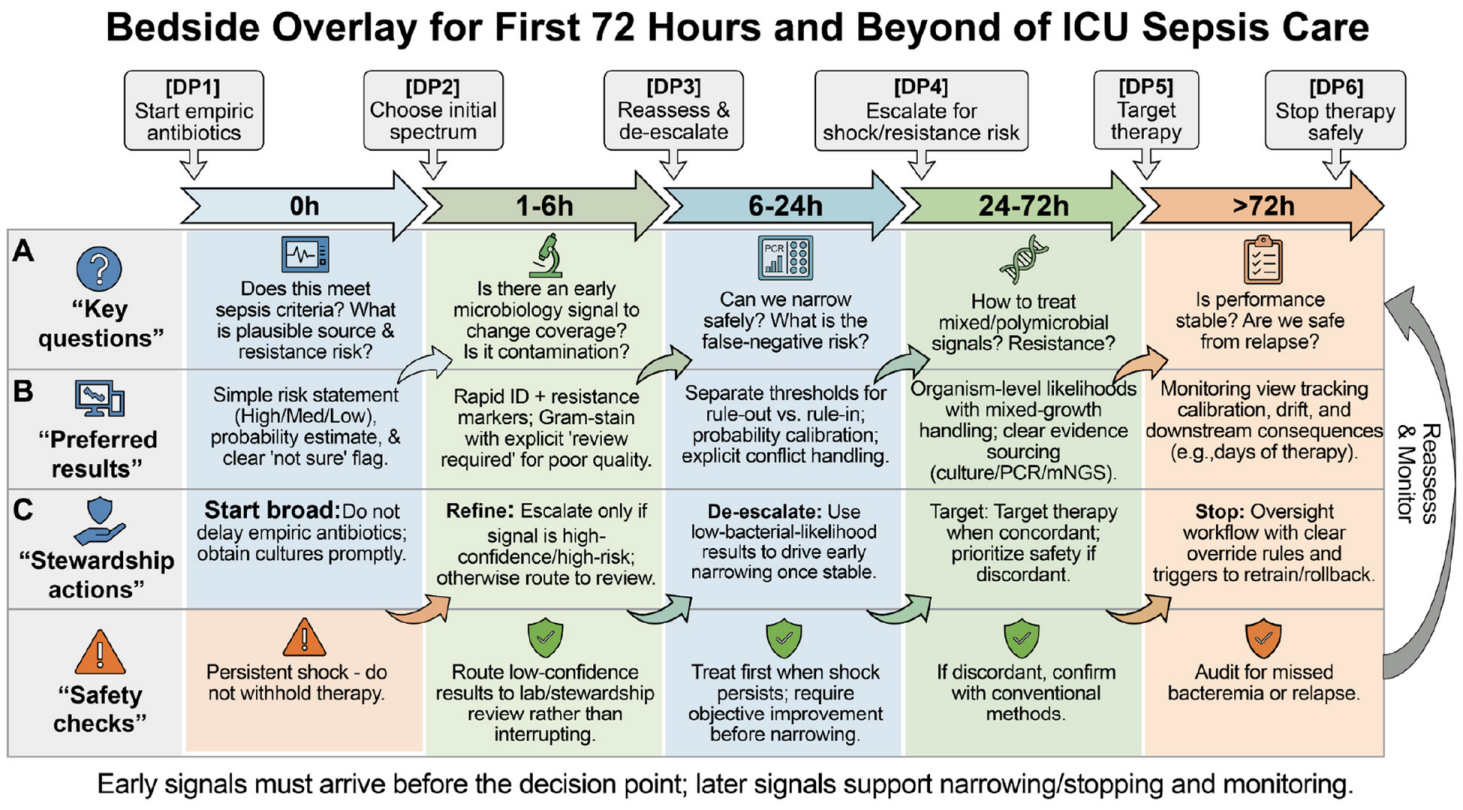
Suggested bedside overlay for ICU sepsis across the first 72 h and beyond, showing decision points, preferred result formats, stewardship actions, and safety checks.

AI tools require ongoing surveillance after implementation because performance can shift as patient populations, local pathogens, and laboratory workflows change. Routine audits should report overall performance and performance in key subgroups (for example, immunosuppressed patients and dialysis patients), and models should be locally validated before transfer to new settings. Fairness frameworks and bias-risk checklists can help teams assess and mitigate inequitable performance before and after clinical launch (Chen et al., 2024a; Chakradeo et al., 2025; Wang et al., 2024). Beyond tool metrics, safety monitoring should include patient-relevant outcomes such as missed bacteremia and relapse, so faster reporting does not compromise clinical safety. Table 4 summarizes practical turnaround targets and preferred result formats by decision point, and Figure 4 provides a bedside overlay for the first 72 hours.

**Table 4:** ICU sepsis-focused turnaround requirements and preferred result formats by decision point.

Decision point (timeline)	Clinical question	Preferred results	Typical action workflow	Examples/evidence
0–1 h: Decide to treat and how broad	Does this meet sepsis criteria? What is the plausible source and resistance risk?	A simple risk statement (High/Med/Low) with a probability estimate and a clear 'not sure' flag; key drivers written in plain language.	Do not delay empiric antibiotics when sepsis is likely; obtain cultures promptly; document the decision point and planned reassessment window.	(Singer et al., 2016; Evans et al., 2021; Levy et al., 2017; Kumar et al., 2006; Peri et al., 2024).
1–6 h: First refinement	Is there an early microbiology signal that should change coverage now? Is it contamination?	Rapid ID + resistance markers; Gram-stain/plate-image prioritization with an explicit 'review required' state for low-quality specimens.	Escalate only when signal is high-confidence/high-risk; otherwise route to lab/stewardship review rather than interrupting bedside flow.	(Signoroni et al., 2023; Bard et al., 2024; Faron et al., 2020; Wang et al., 2025; Curren et al., 2022).
6–24 h: Rule-out vs. Rule-in	Can we narrow safely? What is the false-negative risk for high-harm classes?	Separate thresholds for ruling out vs. ruling in; show probability calibration; explicit conflict handling when physiology disagrees with the tool.	Treat first when shock persists; use low-bacterial-likelihood results to drive early de-escalation once objective improvement and microbiology support it.	(Sounderajah et al., 2025; Moons et al., 2025; Vickers and Elkin, 2006; Vasey et al., 2022).
24–72 h: Targeted therapy	How to treat mixed growth or polymicrobial signals?	Organism-level likelihoods with mixed-growth handling; clear reporting of which evidence supports each suggestion (culture, PCR, mNGS).	Target therapy when concordant; if discordant, prioritize patient safety and confirm with conventional methods and stewardship review.	(Timbrook et al., 2017; Barlam et al., 2016; Wilson et al., 2019; Rivera et al., 2020).
>72 h: Monitoring and safety	Is performance stable? Are we safe?	A monitoring view that tracks probability calibration, drift, and downstream consequences (days of therapy, spectrum score, time to appropriate therapy, safety events).	Oversight clinical workflow with clear override rules, audit cadence, and triggers to retrain or roll back when performance decays.	(Vasey et al., 2022; Group, 2025; Abdelwanis et al., 2024; Chen et al., 2024a; Chakradeo et al., 2025; Wang et al., 2024).

## Clinical vignettes: how multimodal signals can change decisions

### Shock at admission with unclear source

A 62-year-old is transferred from the ward with hypotension, rising lactate, and a new vasopressor requirement. Two blood-culture sets are drawn and empiric antibiotics are started, but bedside ultrasound does not reveal a clear source. The first ICU decision point is already past: treat now, then plan the earliest safe opportunity to refine.

Within the first hour, the laboratory reports a positive blood-culture flag and shares a Gram-stain image. AI-assisted interpretation can help here if it is explicit: the predicted morphology, the confidence, and whether the image quality or mixed signal makes the result unreliable. When the signal is high-confidence Gram-negative rods and the specimen looks clean, it supports early narrowing toward Gram-negative coverage and earlier discussion about resistant-risk features rather than waiting for overnight plate reads (Shwetha et al., 2025; Signoroni et al., 2023; Bard et al., 2024). Later in the first day, a rapid blood-culture molecular panel identifies Enterobacterales and a carbapenemase gene (Wang et al., 2025; Curren et al., 2022). That combination is actionable: it triggers an urgent stewardship review and earlier escalation to an agent with likely activity, before full susceptibility results return. In real life, speed is only an advantage when the result reads as trustworthy. A rapid report should make its confidence visible-mixed morphology, poor fields, or a ‘review required' flag-so the team knows when to act now and when to treat the result as a prompt for human review.

### Neuro ICU fever after hemorrhage: infection or inflammation

A 48-year-old with subarachnoid hemorrhage develops fever, leukocytosis, and increasing oxygen requirements. The chest radiograph is unchanged. The ICU team considers ventilator-associated pneumonia, but also recognizes neurogenic fever and sterile inflammation as competing explanations. In this setting, host-response approaches are most useful as reassessment evidence rather than a single rule-in test. If a validated classifier suggests a low likelihood of bacterial infection, and the patient improves with supportive care and objective safety checks, the team can narrow or stop earlier while documenting a clear ‘restart' plan if deterioration occurs. A ‘low bacterial likelihood' result is not a permission slip to ignore shock. In high-risk physiology, treat first. Where the signal helps is later-when the patient stabilizes and objective data are concordant-and supports earlier, safer de-escalation with a documented rescue plan (Shean and Greninger, 2019; Scicluna et al., 2025; Stevens and Harris, 2025).

### Culture negative sepsis in an immunocompromised patient

A 55-year-old with hematologic malignancy arrives in shock after several antibiotic doses given before blood cultures (Wilson et al., 2019; Brenner et al., 2025; Xu et al., 2025). Cultures stay negative. Computed tomography (CT) raises concern for invasive fungal disease, but nothing is definitive. The bedside question is not whether a test is “perfect”. It is whether any early signal is strong enough to justify antifungal coverage while you move quickly toward confirmation.

Plasma microbial cell-free DNA testing can sometimes provide an organism signal within 24-48 hours (for example, a plausible *Aspergillus* call). Used well, it can support earlier antifungal therapy, earlier infectious-diseases consultation, and faster planning for imaging and tissue sampling-especially when prior antibiotics blunt culture yield. A practical safeguard is 'treat and verify': start time-critical therapy, then actively seek confirmation and track objective response. If the trajectory and markers do not fit, reassess rather than anchoring on a single result. This guardrail is particularly important in immunocompromised patients, where the cost of both delay and misattribution is high. When tissue is obtained, digital pathology and slide triage can shorten time to expert review by flagging regions more likely to contain organisms or invasion (Alfasly et al., 2025; Neidlinger et al., 2025; Zhou et al., 2020). In the near term, the clinical gain is faster prioritization of rare but high-stakes findings-not autonomous diagnosis (Wilson et al., 2019; Brenner et al., 2025; Xu et al., 2025).

## Minimum reporting for clinical trust

### What to report So ICU clinicians can trust the results

A study can be methodologically sound and still fail at the bedside. Reports should state the intended decision point, the time window, and the clinical action the output is meant to support (Vasey et al., 2022; Mongan et al., 2020; Park et al., 2024; Group, 2025). At minimum, studies should follow emerging reporting and bias-assessment standards for clinical AI (Sounderajah et al., 2025; Moons et al., 2025). They should define rule-in and rule-out cut-offs, report probability calibration, and explain how the system handles low-quality specimens, mixed growth, and conflicting evidence. The stewardship workflow should be explicit: who receives the result, who acts, who can override, and how disagreements are documented. Post-deployment monitoring is essential; without surveillance for performance drift, claims of clinical value are not credible (Group, 2025).

### What endpoints matter in infection AI

For ICU infection approaches, endpoints should match the clinical goal (Peri et al., 2024). If the goal is earlier effective therapy, report time to appropriate therapy. If the goal is safer narrowing, report de-escalation within 72 hours and antibiotic days. If the goal is fewer missed infections, report false negative audits in high-risk groups. Mortality is important, but it is often too distal and too confounded to be the only endpoint (Vickers and Elkin, 2006).

Clinical readiness can be judged using a small set of minimum requirements: a clearly stated ICU decision point and time window; a prespecified clinical workflow for rule-in versus rule-out results, including who reviews and can override; probability calibration (or at least transparent error rates at the chosen thresholds); prospective evaluation in routine workflow-ideally including a silent run; and patient-centered and stewardship outcomes such as time to appropriate therapy, antibiotic days, and safety events (Sounderajah et al., 2025; Moons et al., 2025; Collins et al., 2025; Rivera et al., 2020; Vasey et al., 2022; Mongan et al., 2020). These expectations align with widely used reporting and evaluation frameworks for clinical AI.

Implementation essentials (minimum workflow requirements) include a named clinical owner (ICU plus stewardship), a turnaround-time target aligned to the decision point, prespecified documentation and rescue rules (including source control and culture adequacy), and an audit plan that tracks both benefits (earlier appropriate therapy and reduced antibiotic days of therapy) and harms (missed bacteremia, relapse, delays, and adverse drug events). Without workflow integration, even accurate tools rarely change antibiotic decisions.

Governance and monitoring should account for drift in ICU case mix, instruments, and resistance ecology over time. Deployed tools therefore need an operational monitoring plan covering performance and calibration checks, subgroup safety signals, turnaround-time adherence, override rates, and adverse events. Institutions should define ownership for model or test updates, version control, and criteria for pausing use if drift or safety concerns are detected.

Human factors and accountability should be designed deliberately. To reduce cognitive load, outputs should be brief, clinically phrased, and routed to a clearly accountable team (e.g., ICU lead plus antimicrobial stewardship) with defined response times. Interfaces should prioritize decision-relevant summaries-risk band, confidence or deferral, and next-step prompts-rather than raw probabilities alone.

Data governance and privacy should be addressed explicitly when AI outputs are integrated into electronic health records. Institutions should document data provenance, access controls, and retention policies, and ensure that any external processing complies with local privacy regulations. Reporting should avoid unnecessary identifiers while still enabling auditing within the clinical team.

Limitations: This review is framework-driven and therefore prioritizes actionability over exhaustive study enumeration. Evidence quality varies across modalities, ICU populations are heterogeneous, and real-world impact is strongly confounded by turnaround time and co-interventions (source control, catheter removal). Many tools still lack multicenter external validation and prospective impact evaluation, especially in high-risk subgroups (e.g., severe immunosuppression). Generalizability and equity considerations also matter: feasibility, turnaround time, and benefit may differ in resource-limited settings; therefore implementation studies should report local workflow constraints and costs alongside clinical and stewardship outcomes.

## Conclusions and future directions

In day-to-day ICU practice, this review is best used as a 72-hour clinical decision map, not a catalog of tools. At each decision point, clinicians and laboratories can ask three pragmatic questions: (1) Is the signal clinically credible? (2) Could it change management? (3) Does it arrive early enough to matter? AI outputs should be treated as decision-support evidence rather than a verdict. High-stakes actions require confirmatory steps, explicit thresholds for escalation, and a rescue plan when bedside findings conflict with algorithmic suggestions. In the end, value is determined by workflow: who receives the result, how quickly action follows, and what is documented when clinicians appropriately override the tool.

AI for infection in the ICU is most useful when it is designed around decisions, timing, and stewardship (Peri et al., 2024). In the near term, the most actionable approaches surface reliable signal that already exists but is hard to scale (microscopy and culture-plate interpretation) and make complex rapid molecular results easier to interpret under time pressure (Smith et al., 2018; Signoroni et al., 2023; Wang et al., 2025). Larger, more general systems may expand capability-particularly in digital pathology and multimodal integration-but adoption will still hinge on fundamentals: probabilities that match reality, clear confidence communication, safe deferral when uncertainty is high, and transparent pathways for review and override (Vasey et al., 2022; Group, 2025; Huang et al., 2024; Chen et al., 2024b; Lu et al., 2025).

To distinguish this review from prior summaries, we emphasize three takeaways. First, evaluate tools by decision points and time windows, not by aggregate accuracy alone. Second, treat microbiology as longitudinally accruing evidence that can guide therapy over the first 72 hours (Bard et al., 2024; Trigueiro et al., 2024; Mencacci et al., 2023). Third, judge impact in stewardship terms-appropriate initiation, escalation, de-escalation, and duration-alongside diagnostic performance. When studies adopt this clinical framing, it is easier to assess whether a tool supports safe, measurable stewardship gains. Implementation should also be treated as evidence-producing work: laboratories that pair automation with workflow redesign can shorten turnaround time and improve productivity, and recent reviews describe where automation and AI add value and where lab-to-bedside deployment still fails (Trigueiro et al., 2024; Mencacci et al., 2023).
